# Development of a Methodology for Estimating the Ergosterol in Meat Product-Borne Toxigenic Moulds to Evaluate Antifungal Agents

**DOI:** 10.3390/foods10020438

**Published:** 2021-02-17

**Authors:** Micaela Álvarez, Alicia Rodríguez, Elena Bermúdez, Elia Roncero, María J. Andrade

**Affiliations:** 1 Food Hygiene and Safety, Meat and Meat Products Research Institute, Faculty of Veterinary Science, University of Extremadura, Avda. de las Ciencias, s/n. 10003 Cáceres, Spain; maalvarezr@unex.es (M.Á.); bermudez@unex.es (E.B.); eroncerob@unex.es (E.R.); 2 Food Quality and Microbiology, University Institute for the Research in Agrifood Resources, School of Agricultural Engineering, University of Extremadura, Avda. Adolfo Suárez, s/n. 06007 Badajoz, Spain; aliciarj@unex.es

**Keywords:** antifungals, meat products, toxigenic moulds, ergosterol, HPLC-FLD/DAD

## Abstract

Antifungal agents are commonly used in the meat industry to prevent the growth of unwanted moulds, such as toxigenic ones, on dry-cured meat products. For enhancing the application of antifungals, their mode of action must be evaluated. Their effect on the mould ergosterol content is one of the most studied ones, since it is the target site of some commercialised antifungals or of those that are in development. The aim of this study was to develop a methodology for determining how the antifungal agents used in the meat industry work. A method for analysing ergosterol was firstly developed using high-performance liquid chromatography with fluorescence detection coupled to a diode array detector (HPLC-FLD/DAD). The chromatographically optimised conditions (gradient and mobile phases) allowed us to reduce the time per analysis with respect to previously published methods up to 22 min. Withing the six checked extraction methods, method 5, showing the best mean recovery values (99.51%), the shortest retention time (15.8 min), and the lowest standard deviation values (9.92) and working temperature (60 °C), was selected. The limit of detection and limit of quantification were 0.03 and 0.1 µg/mL, respectively. All the validation parameters corroborated the method’s suitability. Finally, its feasibility for evaluating the effect of a commercial antifungal preparation (AP) and different herbs that are frequently added to meat products on the ergosterol content of several toxigenic moulds was studied. Differences at the strain level were obtained in the presence of AP. Moreover, the addition of herbs significantly reduced the ergosterol content in *Penicillium nordicum* up to 83.91%. The developed methodology is thus suitable for screening the antifungals’ role in altering mould ergosterol biosynthesis before their application in real meat products.

## 1. Introduction

Toxigenic moulds are frequently found on the surface of a wide range of dry-cured meat products [1,2]. The concern associated with this kind of moulds is linked to their capability to produce mycotoxins like aflatoxins, ochratoxin A (OTA), or cyclopiazonic acid (CPA) [1,3,4]. Examples of their toxicity are the immunosuppression and liver cancer caused by aflatoxins [5], the nephrotoxicity and the carcinogenicity provoked by OTA [6], and the immunotoxicity on human cells due to CPA [7].

The presence of these unwanted moulds has necessitated the development of several methods to avoid mycotoxin production. The most recently described strategies in dry-cured meat products are related to biocontrol agents employing essential oils [8] or microorganisms, such as yeasts, lactic acid bacteria, and non-toxigenic moulds [9,10,11]. Nutritional and environmental factors greatly affect the growth of the protective cultures, which is a critical drawback for their application. Extensive studies of implementation and viability of such cultures in each particular case are thus necessary [11,12]. Due to such difficulties finding a universal biocontrol agent, the meat industry commonly employs legally authorised antifungals, such as benzoic acid, potassium sorbate (PS), and natamycin [13]. The recently developed alternatives to such synthetic preservatives are based on applying natural compounds of plant origin, such as essential oils or spices, in dry-cured fermented sausages [14,15].

To enhance an antifungal treatment, its mode of action has to be studied before its application. Despite the fact that this aspect has not been fully clarified, several reports have proposed ergosterol as the target site of their activity. Ergosterol is a lipid of the cellular membrane of moulds, which is essential for the growth of their mycelium [16]. Natamycin, one of the most applied antifungals in the meat industry, has been described as blocking mould growth by binding specifically to ergosterol [17]. Similarly, essential oils from oregano and cinnamon have been reported to reduce the ergosterol content of *Fusarium* spp. in maize samples [18]. *Eremanthus erythropappus* essential oil has been also described as a reducer of ergosterol content in *Aspergillus* spp. [19]. Moreover, compounds from essential oils, such as thymol and citral, have shown their effect on ergosterol content in *Fusarium gramineareum* and *Alternaria alternata*, respectively [20,21]. Nevertheless, there is no data about the targets of antifungals of plant origin when utilised against toxigenic moulds in dry-cured meat products.

Ergosterol is not water soluble, but appropriately dissolves in acetone and ethanol [22]. Accordingly, different kinds of organic solvents like butanol and hexane have been used for ergosterol extraction [23,24]. The analytical methods for its detection have been usually based on high-performance liquid chromatography (HPLC), with methanol as eluent [23,24,25], or gas chromatography (GC) with mass spectrometry detection [26]. Concretely, HPLC coupled with a UV detector is the most used method for analysing ergosterol [27,28], since it is easy to handle and has lower maintenance costs and technical requirements than other detectors or chromatographic techniques [29,30]. Whatever the method chosen, it is necessary to consider that the loss of ergosterol due to decomposition and light sensitivity can occur because of the time required for the different steps of the procedure [31]. Therefore, a reduction of 43% of its content after 24 h has been found under light action [25]. Due to the fact that the use of GC for ergosterol quantification implies previous sample derivatisation [26], such a technique could thus negatively affect the results and collaterally increase the time and the cost per analysis. For all the above-mentioned reasons, it is required that a faster and more convenient method be developed for checking the effect of antifungals on mould ergosterol content.

The aim of this study was to develop a methodology for evaluating the impact of antifungal agents on the ergosterol content of meat product-borne toxigenic moulds. Thus, a method for analysing ergosterol using HPLC with fluorescence detection coupled to a diode array detector (HPLC-FLD/DAD), as well as an extraction procedure based on previously published literature, were firstly optimised. After validating both protocols, their viability to evaluate the effect of a commercial antifungal preparation (AP) and different herbs frequently added to cured meat products on the ergosterol content of several toxigenic moulds was evaluated.

## 2. Materials and Methods

### 2.1. Standard and Reagents

All the chemical and chromatographic reagents were HPLC or analytical grade. Acetone, NaOH, 1-butanol, and chloroform were purchased from Scharlab, S.L. (Barcelona, Spain). Hexane, methanol, and acetic acid were obtained from Fisher Scientific S.L. (Hampton, VA, USA). Toluene was purchased from Panreac Química S.A. (Barcelona, Spain), and ergosterol ≥95% HPLC grade from Sigma-Aldrich (San Luis, MO, USA).

The AP containing natamycin and PS was supplemented with 60 g/L of NaCl (Fisher Scientific S.L.), following the manufacturer’s instructions (Manufacturas Taberner, S.A., Valencia, Spain).

### 2.2. Preparation of Standard Solutions

A stock solution (1 mg/mL) was prepared by dissolving standard ergosterol in acetone following the manufacturer’s recommendations. Working solutions were daily prepared in limited light conditions using amber vials by diluting the standard solution in methanol, since it was the main constituent of the mobile phase (Cosela S.L., Sevilla, Spain). They were filtered through a 0.22 µm pore size nylon membrane (Cosela S.L.).

### 2.3. HPLC Method

Ergosterol was analysed by a HPLC-FLD/DAD model 1260 Infinity (Agilent Technologies, Santa Clara, CA, USA). The column was a Phenomenex Luna C_18_, (250 × 4.6 mm, 5 µm particle size; Phenomenex, Macclesfield, United Kingdom). The column temperature was established at 25 °C. The flow rate was set at 1 mL/min, and the injection volume was 20 µL. Ergosterol was detected at λ = 282 nm by the DAD and identified by the absorption spectrum of the standard. Calibration curves were built by diluting the stock solution from 0.1 to 100.0 µg/mL.

To optimise the analytical method for detecting and quantifying ergosterol, the gradients and composition of the mobile phase were assessed using the standard solutions. Firstly, 10 different gradients of mobile phase 1 (MP1), consisting of methanol/acetic acid 0.05% (*v*/*v*) 90/10 (*v*/*v*) (eluent A [24]) and methanol (eluent B) were evaluated (Table S1, Supplementary Materials).

After selecting the most appropriate gradient, three mobile phases were checked: MP1; mobile phase 2 (MP2), consisting of methanol/acetic acid 0.1% (*v*/*v*) 90/10 (*v*/*v*); and mobile phase 3 (MP3), composed by methanol/acetic acid 0.05% (*v*/*v*) 95/5 (*v*/*v*). The selection of the most adequate gradient and mobile phase relied on obtaining a chromatographic peak with a narrow width and symmetric shape, showing the earliest retention time and the shortest run time.

To evaluate the ability of the HPLC-FLD/DAD method to study the effect of antifungal agents, the ergosterol standard (10 µg/mL) was incubated in the presence of three AP concentrations (10%, 50%, and 90%) at different incubation times (0, 8, and 24 h). These concentrations represented different potential quantities of the AP found in meat products: 10% being a low level of the AP, 50% as a medium level, and 90% as a high level. Samples without AP (0%) were used as positive controls.

### 2.4. Extraction of Ergosterol

A total of six methods for extracting mould ergosterol were evaluated. They consisted of two previously reported ones [23,24] and modifications of such methods (Table 1).

The accuracy of each method was evaluated by the percentages of recovery (absolute recoveries) and the method with the best results (≈100% of recovery) was selected. The neat solvent was spiked by adding 10 µg/mL of ergosterol and then extracted following the above-mentioned methods. The results were compared with the area of a standard at the same concentration (10 µg/mL). The percentage of recovery (*RA*) was calculated according to the following equation:

(1) $$RA\;(\%)=\frac{\mathrm{average}\;\mathrm{area}\;\left(\mathrm{samples}\right)}{\mathrm{average}\;\mathrm{area}\;\left(\mathrm{standards}\right)}\times 100$$

All samples were tested in triplicate.

### 2.5. Validation Assays

The HPLC method was validated by determining the limit of detection (LOD) and limit of quantification (LOQ), linearity, precision, repeatability, within-lab reproducibility, and accuracy [7].

The LOD and LOQ were calculated as the minimum ergosterol concentration level at which the signal exceeded the noise level by a factor of 3 (signal-to-noise) and 10, respectively.

Linearity was determined by applying the coefficient of determination (*R*^2^) in the range 0.1–100.0 µg/mL, and the precision by the analysis of three extractions was determined from the same samples under specified conditions. Both parameters were tested in triplicate.

The intra-day repeatability was analysed by three calibration curves in the range 0.1–100.0 µg/mL, conducted by the same operator in duplicate. The inter-day repeatability was checked by three calibration curves performed on three different working days. The within-lab reproducibility was conducted by two different operators, who built three independent calibration curves.

The accuracy was determined by *RA*, as explained in Section 2.4.

### 2.6. Mould Assessment

**Figure 1:**
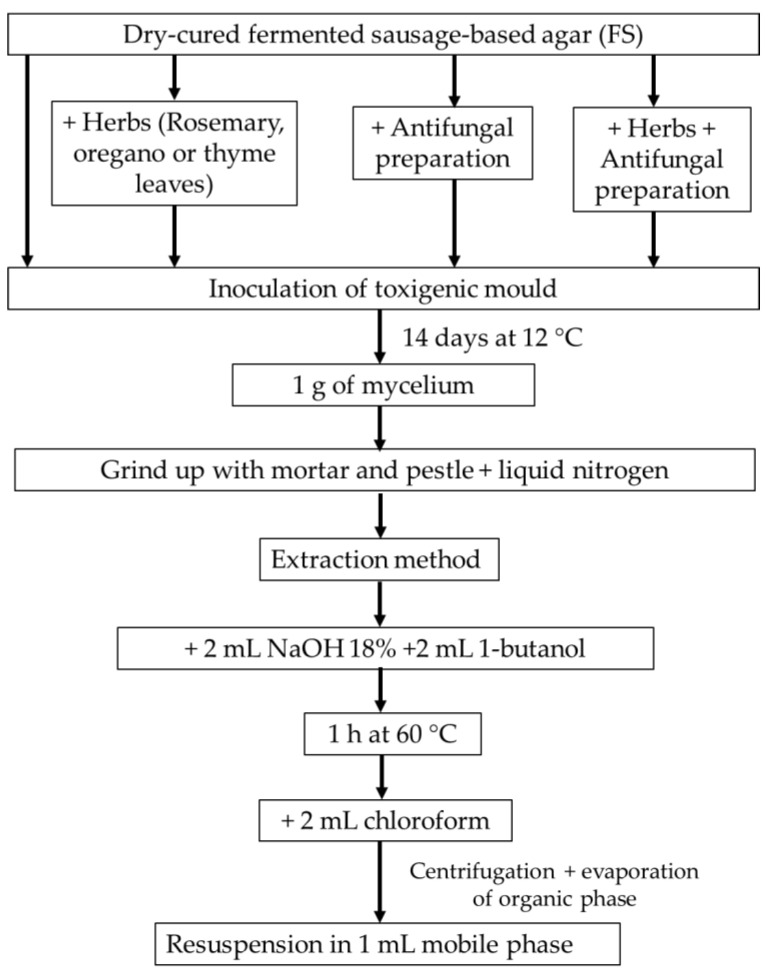
Flow chart showing the assay to check the suitability of the developed methodology for extracting and quantifying ergosterol when determining the effect of antifungal agents against meat product-borne toxigenic moulds. Mobile phase was composed of methanol/acetic acid 0.05% (*v/v*) 95/5 (*v/v*).

To check the suitability of the developed methodology for extracting and quantifying ergosterol when determining the modes of action of antifungal agents, four toxigenic mould strains were incubated in the presence of AP: the ochratoxigenic *Penicillium nordicum* CBS 323.92, belonging to Centraalbureau voor Schimmelcultures (The Netherlands), and *P. nordicum* BFE 856 from the Federal Research Centre for Nutrition and Food (Germany); the aflatoxigenic *Aspergillus flavus* CBS 573.65 from Centraalbureau voor Schimmelcultures; and the CPA producer *Penicillium griseofulvum* IBT 14319, supplied by the Type Culture Collection of the Department of Biotechnology from the Technical University of Denmark. In addition, *P. nordicum* CBS 323.92, one of the main OTA producers in meat products, was also selected to analyse the ergosterol content after applying rosemary, oregano, and thyme, which are herbs recently described as having antifungal activity in dry-cured fermented sausages [15] (Figure 1).

The mould inocula were prepared by growing them on potato dextrose agar (PDA; Scharlab, S.L.) at 25 °C for 7 days. Spores were harvested by scraping the plate surface with a glass rod after adding 3 mL of phosphate-buffered saline (0.32 g of NaH_2_PO_4_ (Scharlab, S.L.), 1.09 g of Na_2_HPO_4_ (Scharlab, S.L.), 9 g of NaCl (Scharlab, S.L.), and 1 L of distilled water). Spore suspensions were quantified using a Thoma Blaubrand counting chamber (Brand, Germany). A concentration of 10^6^ spores/mL of each mould strain was individually inoculated on a dry-cured fermented sausage-based agar (FS; [15]). A volume of 25 µL of AP was distributed on the surface of the FS before the mould inoculation. To test the herbs, the FS was supplemented with 2 g/kg of rosemary (FS-R), oregano (FS-O), and thyme (FS-T), as described by Álvarez et al. [15]. Fresh rosemary and thyme leaves were used, while oregano leaves were added dried. The herbs were harvested in the region of Extremadura, located in the southwest of Spain. The effect of the herbs on the mould ergosterol content was evaluated alone and in combination with AP. As negative and positive controls, *P. nordicum* CBS 323.92 grown without herbs and in the presence of AP was used, respectively. The assay was performed in triplicate. After incubating for 14 days at 12 °C, 1 g of the mycelia was collected by scraping the surface with a scalpel and frozen with liquid nitrogen to stop the metabolic activity of the moulds before storing them at −80 °C. For the ergosterol extraction, the samples were firstly homogenised using a mortar and pestle with liquid nitrogen, and the obtained mycelium powder was subjected to the selected extraction method (Figure 1).

### 2.7. Statistical Analysis

Data analysis was carried out using the SPSS v. 20 software (IBM Corporation, Armonk, NY, USA). The non-parametric Kruskal–Wallis and Mann–Whitney U tests were applied, since the data failed the Shapiro–Wilk and Levene tests. The statistical significance was established at *p* ≤ 0.05. The standard deviations were determined using Microsoft Excel 365 (Microsoft, Albuquerque, NM, USA).

## 3. Results and Discussion

### 3.1. Improvement of the Chromatographic Conditions

A reversed-phase column (C_18_) was selected for the HPLC-FLD/DAD detection method, due to its good peak resolution [23,27]. Firstly, the selection of the most appropriate mobile phase was performed (Table S1, Supplementary Materials). Gradient 10 was the most suitable, since its retention time was the earliest (17.43 min) and its run time the shortest (22 min). Gradient 8 gave similar results, but it was dismissed because of its longer run time (27 min). The chosen gradient managed to reduce the analysis time to 45% compared to the previously developed methods for ergosterol detection, which employed between 30 and 40 min per analysis [23,24,32]. Such a method would thus allow minimising the potential degradation of ergosterol because of light exposure, with a subsequent decrease in false-negative results.

**Figure 2:**
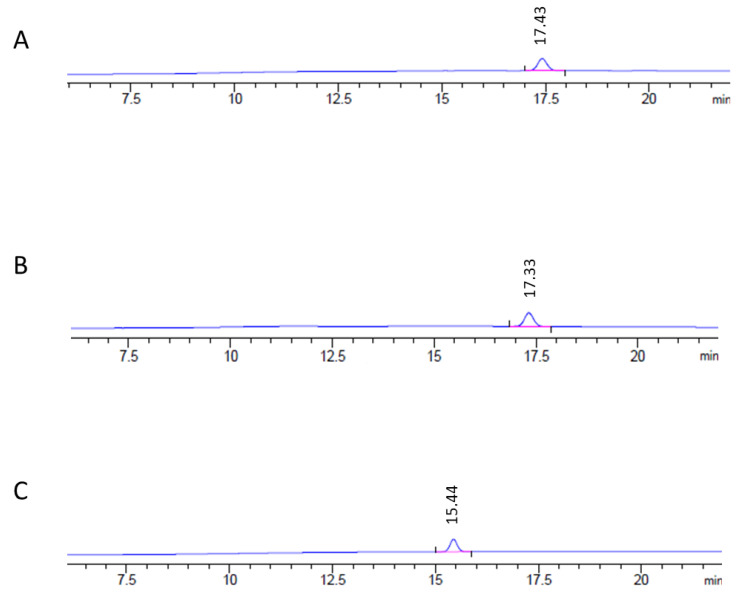
High-performance liquid chromatography with fluorescence detection coupled to a diode array detector (HPLC-FLD/DAD) chromatograms using an ergosterol standard (10 µg/mL) and the three different mobile phases tested in the present work. (**A**) Mobile phase 1, composed of methanol/acetic acid 0.05% (*v*/*v*) 90/10 (*v*/*v*); retention time: 17.43 min. (**B**) Mobile phase 2, composed of methanol/acetic acid 0.1% (*v*/*v*) 90/10 (*v*/*v*); retention time: 17.33 min. (**C**) Mobile phase 3, composed of methanol/acetic acid 0.05% (*v*/*v*) 95/5 (*v*/*v*); retention time: 15.44 min. Eluent A was composed of the corresponding mobile phase, and Eluent B of methanol. The gradient consisted of 0–5 min 10% B, 5–8 min linear increase from 10% to 100% B, 8–12 min 100% B, 12–22 min linear decrease from 100% to 10% B, with methanol being used as eluent B.

Regarding the mobile-phase composition, MP3, containing less acetic acid than MP1 and MP2, considerably reduced the retention time without losing precision, as its coefficient of variation value (7.59%) was less than 15% (Table 2). This is considered adequate by different official agencies [33,34]. MP3 also showed the best results for the peak width and asymmetry values (Table 2). A symmetrical, sharp peak with a narrow width (asymmetry value close to 1) has higher precision and is better separated than a more curved peak [35,36]. Such differences between mobile phases are shown in the chromatograms included in Figure 2. The gradient and mobile phase improvement was thus associated with shorter analysis time, enabling increased laboratory productivity because of the increase of the amount of analysis per day and the reduction of reagent costs.

When the ability of the HPLC-FLD/DAD method to study the mode of action of antifungal agents was evaluated in the presence of different amounts of AP, a lack of detection of ergosterol was observed in the presence of the highest amount (Table 3). These findings corroborate those previously reported for both AP’s components (PS and natamycin) when evaluating their effect on mould ergosterol biosynthesis. Therefore, PS has been associated with changes in the lipid composition, decreasing the sterol content [37] with the resultant reduction in ergosterol, the principal sterol in moulds [26]. In contrast, natamycin specifically binds to ergosterol, blocking mycelial growth [17]. In the present work, interference between AP and ergosterol depended on the applied concentration and not on the exposition time, which is of crucial interest for the meat industry, since the use of sub-inhibitory doses of antifungals has been related to undesirable mould growth, the emergence of mould resistance, and mycotoxin production [38,39,40].

### 3.2. Extraction Method

When searching for reductions in the analysis time, the extraction method is considered to be as important as the detection one. The results obtained when evaluating the six different methods for extracting ergosterol are shown in Table 1. When testing the two previously reported methods (Methods 1 and 2), the lowest recoveries values were obtained. In addition, Method 2 presented a high standard deviation, which could be due to the fact that this method was originally designed to be used in yeast [23] instead of filamentous fungi that have a more complex cell wall. On the contrary, the best mean recovery values, together with the shortest retention times, were shown by Methods 3 and 5. The saponification step, with a higher concentration of NaOH in Methods 2 to 6, seems to be crucial for ergosterol extraction by increasing cell wall permeability and facilitating the entry of alcohols [23]. This feature, as well as the use of methanol instead of 1-butanol for extraction, could be the reason for the low recovery value found in Method 1. This is in accordance with Pastinen et al. [23], who described that alcohols with four carbons, such as 1-butanol, are better solvents for ergosterol extraction than alcohols with fewer carbons like methanol, which was that employed in Method 1.

Furthermore, when comparing Method 2 and those developed from it (Methods 3–6), it appears that the addition of chloroform instead of toluene triggers the improvement of the recoveries. This could be due to the good solubility of ergosterol in chloroform [41], which might also help to increase the permeability of the cell wall. Additionally, the permeability could increase by prolonging the incubation time, as better results were obtained when samples were incubated for 1 h (Method 3) than for 30 min (Method 4).

Within the procedures with the best outcomes, Methods 3 and 5 were the most appropriate. Method 5 was finally selected because of its lower standard deviation values and temperature than Method 3, since the detection of ergosterol can be negatively affected by high temperatures [42]. Consequently, Method 5 has been able to improve the results obtained from the previously reported methods [23,24].

### 3.3. Validation Parameters

The values for LOD and LOQ were 0.03 and 0.1 µg/mL, respectively, lower than those previously obtained by other authors using HPLC-FLD [24,30,42].

**Figure 3:**
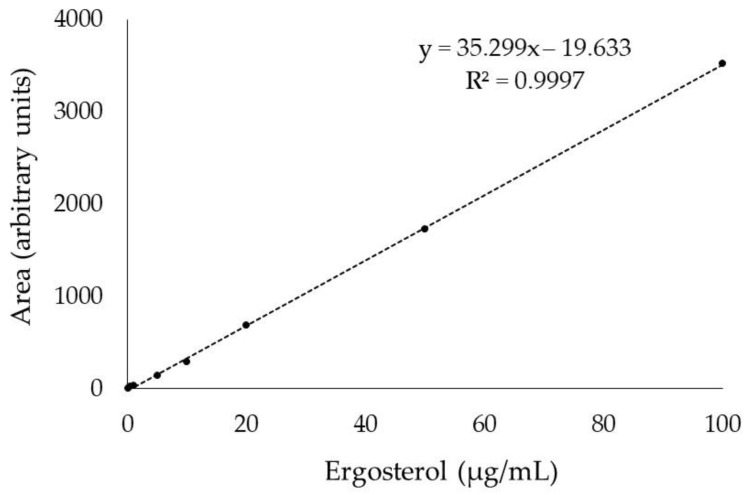
Standard calibration curve built in the range 0.1–100.0 µg/mL of ergosterol using the optimised HPLC-FLD/DAD method.

The linearity defined by calibration lines relating to ergosterol concentration and peak area gave an optimum value (Figure 3). The *R*^2^ value is considered an indicator of linearity, and values close to the unit are synonymous to a straight line [29,43], while some studies have demonstrated curved relationships and suggest a visual inspection of calibration plots to confirm the linearity [44,45]. In our study, the linear trend of the calibration line is clearly visualised in Figure 3, supporting the good linearity of the method.

The calibration curves for intra-day and inter-day repeatability were similar, and demonstrated no significant differences between them, like the ones used for within-lab reproducibility (*p* ≤ 0.05) (data not shown). These features support the use of the method independently of the operator.

### 3.4. Ergosterol Content of Toxigenic Moulds in the Presence of Antifungal Compounds

The extraction and HPLC-FLD/DAD methods were finally used to evaluate the effect of the AP and the selected herbs on the ergosterol content of toxigenic moulds (Table 4 and Table 5). It should be pointed out that the assay was carried out using a meat-based model and not a real meat matrix, since the presence of the native microbial population of the latter having ergosterol would not give feasible results about the effect of the antifungals on the content of such compound. Nonetheless, a full understanding of the targets of antifungal strategies is desirable, since it would allow the establishment of the best conditions for their addition, minimising mycotoxin exposure, and consequently, improving food safety. Accordingly, FS has been selected to perform this assay, due to the fact that this culture medium efficiently simulates the composition of dry-cured, fermented sausages [15].

On the other hand, the novelty of the method applied with moulds has to be highlighted. The method consists of blocking the moulds’ metabolic activity after sampling using liquid nitrogen (Figure 1), thereby reducing ergosterol instability following the mould death.

It has been described that ergosterol production depends on the mould species and colony age, as well as on the substrate [46]. Accordingly, our results show that *Penicillium* species were able to produce higher ergosterol amounts than *A. flavus* under control conditions. To our knowledge, there are no studies about mould ergosterol levels in a meat-based substrate, but previous studies have demonstrated a higher production of ergosterol in Penicillia than in Aspergilla in PDA and rice [46,47]. Furthermore, the influence of the AP on the ergosterol content in the tested toxigenic Penicillia moulds was corroborated, because it was significantly reduced in those samples incubated in its presence. The reducing effect of the components of AP on ergosterol content has been previously reported in Penicillia. Thus, PS has been described as significantly reducing the sterol content in *Penicillium roqueforti*, affecting the lipid bilayer [37], while natamycin has shown a high binding to ergosterol during germination in *Penicillium discolor* [48]. Additionally, ergosterol has shown to be the target of antifungal drugs used to treat fungal infections, such as azoles, which inhibit ergosterol biosynthesis in *Aspergillus fumigatus* [49].

On the contrary, the ergosterol content of *A. flavus* CBS 573.65 was not affected in the presence of the AP in this study. This lack of effect of natamycin on mould ergosterol has been previously described in *Aspergillus ochraceus*, and has been associated with the existence of resistances due to its continuous use; it even could enhance resistance to other polyene antifungals by cross-tolerance [39].

Regarding the effect of the addition of rosemary, oregano, and thyme leaves against the ochratoxigenic *P. nordicum* CBS 323.92, all of them significantly reduced the ergosterol content regardless of whether they were in the presence or absence of AP (Table 5). These findings are in accordance with previous studies testing different essential oils against toxigenic moulds. Thus, it has been reported that the essential oil extracted from rosemary decreased in vitro ergosterol content in toxigenic *A. flavus* and *Fusarium verticillioides*, although the minimum inhibitory concentration differs depending on the species [50,51]. Regarding oregano and thyme essential oils, some of their main components, such as carvacrol or thymol, have been reported to diminish the ergosterol content in *A. flavus* [52]. Despite the fact that there are not many studies focused on Penicillia, some essential oils, such as tea tree oil and betel leaf oil, have shown ergosterol reductions in *Penicillium expansum* [53,54]. The natural compounds cinnamaldehyde and citral from cinnamon and *Cymbopogon*, respectively, have also shown their effect by decreasing ergosterol in *P. expansum* [55]. Another study has suggested that the compound antofine from *Cynanchum atratum* can significantly impair ergosterol biosynthesis in *Penicillium digitatum* [56].

Therefore, the ergosterol seems to be the target site of the antifungal action of rosemary, oregano, and thyme, which could explain the reduction of OTA presence detected by Álvarez et al. [15] when testing their effects against *P. nordicum*. Accordingly, the synthesis of ergosterol has been associated with the OTA production in *A. ochraceus* and *P. verrucosum* [47,57]. Nevertheless, further studies are necessary to elucidate other modes of action that could be involved in the antifungal activities of rosemary, oregano, and thyme, since synergic effects are generally attributed to strategies against unwanted moulds.

## 4. Conclusions

A new methodology for the extraction and quantification of ergosterol from toxigenic moulds was developed to be used when evaluating the role of antifungal compounds applied in the meat industry. The method turned out to be precise, fast, and effective for ergosterol quantification in meat product-borne moulds. The method improved the previously developed procedures, due to the reduction of the run time up to 22 min, and consequently, the possible degradation of ergosterol in ergocalciferol. In addition, the methodology proved to be useful for determining the effect of antifungal agents related to dry-cured meat products on the ergosterol content, independent of the toxigenic mould strain. The present method could be thus used as a support tool for improving information about antifungals before their application in meat products, which would enhance their use.

## Figures and Tables

**Table 1:** Main procedure steps and results of the methods for extraction of mould ergosterol evaluated in this study.

Methods	Main Steps Involved in the Procedure	Recovery (%)	Standard Deviation (%) ^2^	Retention Time (min)	References
Method 1	2 mL NaOH 10% (*w*/*v*) in methanol + vortex 30 s + 1 h at 60 °C + 2 mL distilled water + 5 mL hexane + evaporation of the hexane extract + resuspension in 1 mL MP ^1^	9.11 *	±6.97	16.7	[24]
Method 2	2 mL NaOH 18% (*w*/*v*) in distilled water + 2 mL 1-butanol + vortex 30 s + 1 h at 90 °C + 2 mL toluene + centrifugation (5000 rpm for 5 min) + evaporation of the organic phase + resuspension in 1 mL MP	47.45 *	±73.69	16.4	[23]
Method 3	2 mL NaOH 18% (*w*/*v*) in distilled water + 2 mL 1-butanol + vortex 30 s + 1 h at 90 °C + 2 mL chloroform + centrifugation (5000 rpm for 5 min) + evaporation of the organic phase + resuspension in 1 mL MP	103.10	±26.97	15.8	This study
Method 4	2 mL NaOH 18% (*w*/*v*) in distilled water + 2 mL 1-butanol + vortex 30 s + 30 min at 90 °C + 2 mL chloroform + centrifugation (5000 rpm for 5 min) + evaporation of the organic phase + resuspension in 1 mL MP	82.12	±80.37	15.9	This study
Method 5	2 mL NaOH 18% (*w*/*v*) in distilled water + 2 mL 1-butanol + vortex 30 s + 1 h at 60 °C + 2 mL chloroform + centrifugation (5000 rpm for 5 min) + evaporation of the organic phase + resuspension in 1 mL MP	99.51	±9.92	15.8	This study
Method 6	2 mL NaOH 18% (*w*/*v*) in distilled water + 2 mL 1-butanol + vortex 30 s + 1 h at 90 °C + 3 mL chloroform + centrifugation (5000 rpm for 5 min) + evaporation of the organic phase + resuspension in 1 mL MP	81.09 *	±21.63	15.9	This study

^1^ MP: mobile phase, composed of methanol/acetic acid 0.05% (*v*/*v*) 95/5 (*v*/*v*). ^2^ The experiment was performed in triplicate. * Significant differences regarding 100% recovery (*p* ≤ 0.05). Ergosterol solutions were prepared from the standard to achieve a final concentration of 10 µg/mL. Different extraction solutions, reagent concentrations, and temperature and incubation times were checked. The extraction was always performed under limited light conditions.

**Table 2:** Results from the evaluation of three mobile phases (MP1–3) for detecting ergosterol (10 µg/mL) with Gradient 10 (0–5 min 10% B ^1^, 5–8 min linear increase from 10% to 100% B, 8–12 min 100% B, and 12–22 min linear decrease from 100% to 10% B).

Mobile Phases ^2^	Retention Time (min)	Coefficient of Variations (%) ^3^	Width of the Peaks (min)	Asymmetry
MP1	17.43	12.75	0.24	0.90
MP2	17.33	8.79	0.23	0.91
MP3	15.44	7.59	0.18	0.97

^1^ Eluent A was composed of the corresponding MP and Eluent B by methanol. ^2^ MP1: methanol/acetic acid 0.05% (*v*/*v*) 90/10 (*v*/*v*); MP2: methanol/acetic acid 0.1% (*v*/*v*) 90/10 (*v*/*v*); MP3: methanol/acetic acid 0.05% (*v*/*v*) 95/5 (*v*/*v*). ^3^ The experiment was performed in triplicate.

**Table 3:** The concentration of ergosterol (µg/mL) when co-inoculated (10 µg/mL) with different amounts of antifungal preparation (AP) at different sampling times.

AP (%, *v*/*v*)	Concentration of Ergosterol		
	0 h	8 h	24 h
0	8.74 ± 1.28 ^1^	8.44 ± 1.60	9.11 ± 0.72
10	9.28 ± 0.71	8.85 ± 0.56	9.03 ± 0.69
50	0.50 ± 0.43 *	n.d *	n.d *
90	n.d ^2^*	n.d *	n.d *

^1^ The experiment was performed in triplicate. ^2^ n.d: not detected (<limit of detection) * Significant differences regarding the absence of AP at the same sampling time (*p* ≤ 0.05).

**Table 4:** Effect of an antifungal preparation (AP) on the ergosterol content (µg/g mycelium) of four toxigenic mould strains.

	Mould Strain			
	*Penicillium nordicum* CBS 323.92	*P. nordicum* BFE 856	*Penicillium griseofulvum* IBT 14319	*Aspergillus flavus* CBS 573.65
Control	731.55 ± 183.54 ^1^	365.57 ± 45.96	1121.61 ± 486.34	248.09 ± 174.49
Mould + AP	513.59 ± 92.84 *	187.15 ± 92.14 *	185.89 ± 158.75 *	297.22 ± 207.47

^1^ The experiment was performed in triplicate. * Significant differences regarding the control (*p* ≤ 0.05).

**Table 5:** Effect of rosemary, oregano, and thyme on the ergosterol content (µg/g of mycelium) of *Penicillium nordicum* CBS 323.92.

Treatment ^1^	Ergosterol Content
FS	731.55 ± 183.54 ^2^
FS-R	184.34 ± 44.50 *
FS-O	177.40 ± 17.50 *
FS-T	117.70 ± 69.30 *
FS-R + AP	132.16 ± 44.50 *
FS-O + AP	204.73 ± 50.85 *
FS-T + AP	237.57 ± 11.99 *

^1^ FS: dry-cured, fermented, sausage-based agar (control); FS-R: FS with rosemary; FS-O: FS with oregano; FS-T: FS with thyme; FS-R + AP: FS-R with antifungal preparation; FS-O + AP: FS-O with antifungal preparation; FS-T + AP: FS-T with antifungal preparation. ^2^ The experiment was performed in triplicate. * Significant differences regarding the control (*p* ≤ 0.05).

## Data Availability

Data is contained within the article or supplementary material.

## Supplementary Materials

The following are available online at https://www.mdpi.com/2304-8158/10/2/438/s1, Table S1: Mobile phase gradient conditions evaluated in this study for detecting and quantifying ergosterol in meat product-borne toxigenic moulds by HPLC-FLD/DAD.
