# Supplementary material for: Development of a Methodology for Estimating the Ergosterol in Meat Product-Borne Toxigenic Moulds to Evaluate Antifungal Agents
Source: Foods. 2021 Feb 17;10(2):438. doi: 10.3390/foods10020438 (PMC7922909; doi:10.3390/foods10020438)
Supplement: Supplementary file 1 [file foods-10-00438-s001.zip › Table 1. ╡lvarez et al..docx]

## Table 1. Main procedure steps and results of the methods for extraction of mould ergosterol evaluated in this study.

| **Methods** | **Main steps involved in the procedure** | **Recovery (%)** | **Standard deviation (%)^2^** | **Retention time (min)** | **References** |
| --- | --- | --- | --- | --- | --- |
| Method 1 | 2 mL NaOH 10% (w/v) in methanol + vortex 30 s + 1 h at 60 ºC + 2 mL distilled water + 5 mL hexane + evaporation of the hexane extract + resuspension in 1 mL MP3^1^ | 9.11* | ±6.97 | 16.7 | Neuhof et al., 2008 |
| Method 2 | 2 mL NaOH 18% (w/v) in distilled water + 2 mL 1-butanol + vortex 30 s + 1 h at 90 ºC + 2 mL toluene + centrifugation 5,000 rpm for 5 min + evaporation of the organic phase + resuspension in 1 mL MP3^1^ | 47.45* | ±73.69 | 16.4 | Pastinen et al., 2017 |
| Method 3 | 2 mL NaOH 18% (w/v) in distilled water + 2 mL 1-butanol + vortex 30 s + 1 h at 90 ºC + 2 mL chloroform + centrifugation 5,000 rpm for 5 min + evaporation of the organic phase + resuspension in 1 mL MP3^1^ | 103.10 | ±26.97 | 15.8 | This study |
| Method 4 | 2 mL NaOH 18% (w/v) in distilled water + 2 mL 1-butanol + vortex 30 s + 30 min at 90 ºC + 2 mL chloroform + centrifugation 5,000 rpm for 5 min + evaporation of the organic phase + resuspension in 1 mL MP3^1^ | 82.12 | ±80.37 | 15.9 | This study |
| Method 5 | 2 mL NaOH 18% (w/v) in distilled water + 2 mL 1-butanol + vortex 30 s + 1 h at 60 ºC + 2 mL chloroform + centrifugation 5,000 rpm for 5 min + evaporation of the organic phase + resuspension in 1 mL MP3^1^ | 99.51 | ±9.92 | 15.8 | This study |
| Method 6 | 2 mL NaOH 18% (w/v) in distilled water + 2 mL 1-butanol + vortex 30 s + 1 h at 90 ºC + 3 mL chloroform + centrifugation 5,000 rpm for 5 min + evaporation of the organic phase + resuspension in 1 mL MP3^1^ | 81.09* | ±21.63 | 15.9 | This study |

^1^MP3: mobile phase composed by methanol/acetic acid 0.05% (v/v) 95/5 (v/v). ^2^The experiment was performed in triplicate. *Significance differences regarding 100% recovery (*P* ≤ 0.05).
