# Supplementary material for: Development of a Methodology for Estimating the Ergosterol in Meat Product-Borne Toxigenic Moulds to Evaluate Antifungal Agents
Source: Foods. 2021 Feb 17;10(2):438. doi: 10.3390/foods10020438 (PMC7922909; doi:10.3390/foods10020438)
Supplement: Supplementary file 1 [file foods-10-00438-s001.zip › Table 2. ╡lvarez et al..docx]

**Table 2.** Results from the evaluation of 3 mobile phases (MP) for detecting ergosterol (10 µg/mL) with the gradient 0-5 min 10% B^1^, 5-8 min linear increase from 10 to 100% B, 8-12 min 100% B and 12-22 min linear decrease from 100 to 10% B.

| **Mobile phases^2^** | **Retention time (min)** | **Coefficient of variations (%)^3^** | **Width of the peaks (min)** | **Asymmetry** |
| --- | --- | --- | --- | --- |
| MP1 | 17.43 | 12.75 | 0.24 | 0.90 |
| MP2 | 17.33 | 8.79 | 0.23 | 0.91 |
| MP3 | 15.44 | 7.59 | 0.18 | 0.97 |

^1^Eluent A was composed by the corresponding MP and Eluent B by methanol. ^2^MP1: methanol/acetic acid 0.05 % (v/v) 90/10 (v/v); MP2: methanol/acetic acid 0.1 % (v/v) 90/10 (v/v); MP3: methanol/acetic acid 0.05 % (v/v) 95/5 (v/v). ^3^ The experiment was performed in triplicate.
