# Supplementary material for: Development of a Methodology for Estimating the Ergosterol in Meat Product-Borne Toxigenic Moulds to Evaluate Antifungal Agents
Source: Foods. 2021 Feb 17;10(2):438. doi: 10.3390/foods10020438 (PMC7922909; doi:10.3390/foods10020438)
Supplement: Supplementary file 1 [file foods-10-00438-s001.zip › Table 3. ╡lvarez et al..docx]

**Table 3.** The concentration of ergosterol (µg/mL) when co-inoculated (10 µg/mL) with different amounts of antifungal preparation (AP) at different sampling times.

| **AP (%, v/v)** | **Concentration of ergosterol** | | |
| --- | --- | --- | --- |
|  | **0 h** | **8 h** | **24 h** |
| 0 | 8.74 ± 1.28^1^ | 8.44 ± 1.60 | 9.11 ± 0.72 |
| 10 | 9.28 ± 0.71 | 8.85 ± 0.56 | 9.03 ± 0.69 |
| 50 | 0.50 ± 0.43* | n.d* | n.d* |
| 90 | n.d^2^* | n.d* | n.d* |

^1^The experiment was performed in triplicate. ^2^n.d: not detected (< Limit of Detection) *Significance differences regarding the absence of AP at the same sampling time (*P* ≤ 0.05).
