# Supplementary material for: Development of a Methodology for Estimating the Ergosterol in Meat Product-Borne Toxigenic Moulds to Evaluate Antifungal Agents
Source: Foods. 2021 Feb 17;10(2):438. doi: 10.3390/foods10020438 (PMC7922909; doi:10.3390/foods10020438)
Supplement: Supplementary file 1 [file foods-10-00438-s001.zip › Table 4. ╡lvarez et al..docx]

**Table 4.** Effect of an antifungal preparation (AP) on the ergosterol content (µg/g mycelium) of four toxigenic mould strains.

|  | **Mould strain** | | | |
| --- | --- | --- | --- | --- |
|  | *Penicillium nordicum* CBS 323.92 | *P. nordicum* BFE 856 | *Penicillium griseofulvum* IBT 14319 | *Aspergillus flavus* CBS 573.65 |
| Control | 731.55±183.54^1^ | 365.57±45.96 | 1121.61±486.34 | 248.09±174.49 |
| Mould+AP | 513.59±92.84* | 187.15±92.14* | 185.89±158.75* | 297.22±207.47 |

^1^ The experiment was performed in triplicate. *Statistical differences regarding the control (*P* ≤ 0.05).
