# Supplementary material for: Development of a Methodology for Estimating the Ergosterol in Meat Product-Borne Toxigenic Moulds to Evaluate Antifungal Agents
Source: Foods. 2021 Feb 17;10(2):438. doi: 10.3390/foods10020438 (PMC7922909; doi:10.3390/foods10020438)
Supplement: Supplementary file 1 [file foods-10-00438-s001.zip › Table 5. ╡lvarez et al..docx]

**Table 5.** Effect of rosemary, oregano, and thyme on the ergosterol content (µg/g of mycelium) of *Penicillium nordicum* CBS 323.92.

| **Treatment** | **Ergosterol content** |
| --- | --- |
| FS  FS-R | 731.55 ± 183.54^1^  184.34 ± 44.50* |
| FS-O | 177.40 ± 17.50* |
| FS-T | 117.70 ± 69.30* |
| FS-R+AP | 132.16 ± 44.50* |
| FS-O+AP | 204.73 ± 50.85* |
| FS-T+AP | 237.57 ± 11.99* |

FS: dry-cured fermented sausage-based agar (control); FS-R: FS with rosemary; FS-O; FS with oregano; FS-T: FS with thyme; FS-R+AP: FS-R with antifungal preparation; FS-O+AP: FS-O with antifungal preparation; FS-T+AP: FS-T with antifungal preparation. ^1^The experiment was performed in triplicate. *Significance differences regarding the control (*P* ≤ 0.05).
