# Supplementary material for: Development of a Methodology for Estimating the Ergosterol in Meat Product-Borne Toxigenic Moulds to Evaluate Antifungal Agents
Source: Foods. 2021 Feb 17;10(2):438. doi: 10.3390/foods10020438 (PMC7922909; doi:10.3390/foods10020438)
Supplement: Supplementary file 1 [file foods-10-00438-s001.zip › Table S1. ╡lvarez et al.docx]

## Table S1. Gradient conditions of the mobile phases evaluated in this study for detecting and quantifying ergosterol in moulds by HPLC-FLD/DAD.

| **Gradients** | **Characteristics** | **Retention time** | **Width of the peaks** | **Asymmetry** |
| --- | --- | --- | --- | --- |
| Gradient 1 | 0-10 min 10% B^1^, 10-15 min linear increase from 10 to 100% B, 15-20 min 100% B, 20-40 min linear decrease from 100 to 10% B | 22.70 | 0.24 | 0.94 |
| Gradient 2 | 0-10 min 10% B, 10-18 min linear increase from 10 to 100% B, 18-23 min 100% B, 23-40 min linear decrease from 100 to 10% B | 24.30 | 0.24 | 0.94 |
| Gradient 3 | 0-10 min 10% B, 10-18 min linear increase from 10 to 100% B, 18-25 min 100% B, 25-40 min linear decrease from 100 to 10% B | 24.31 | 0.24 | 0.92 |
| Gradient 4 | 0-5 min 10% B, 5-15 min linear increase from 10 to 100% B, 15-20 min 100% B, 20-35 min linear decrease from 100 to 10% B | 21.14 | 0.19 | 0.91 |
| Gradient 5 | 0-7 min 10% B, 7-17 min linear increase from 10 to 100% B, 17-22 min 100% B, 22-37 min linear decrease from 100 to 10% B | 22.90 | 0.18 | 0.92 |
| Gradient 6 | 0-7 min 10% B, 7-12 min linear increase from 10 to 100% B, 12-17 min 100% B, 17-32 min linear decrease from 100 to 10% B | 20.24 | 0.19 | 0.90 |
| Gradient 7 | 0-5 min 10% B, 5-10 min linear increase from 10 to 100% B, 10-15 min 100% B, 15-30 min linear decrease from 100 to 10% B | 18.48 | 0.19 | 0.92 |
| Gradient 8 | 0-5 min 10% B, 5-8 min linear increase from 10 to 100% B, 8-12 min 100% B, 12-27 min linear decrease from 100 to 10% B | 17.49 | 0.22 | 0.92 |
| Gradient 9 | 0-5 min 10% B, 5-10 min linear increase from 10 to 100% B, 10-15 min 100% B, 15-25 min linear decrease from 100 to 10% B | 18.30 | 0.20 | 0.91 |
| Gradient 10 | 0-5 min 10% B, 5-8 min linear increase from 10 to 100% B, 8-12 min 100% B, 12-22 min linear decrease from 100 to 10% B | 17.42 | 0.24 | 0.91 |

^1^Eluent B: methanol.
