# Supplementary material for: Development of a Methodology for Estimating the Ergosterol in Meat Product-Borne Toxigenic Moulds to Evaluate Antifungal Agents
Source: Foods. 2021 Feb 17;10(2):438. doi: 10.3390/foods10020438 (PMC7922909; doi:10.3390/foods10020438)
Supplement: Supplementary file 1 [file foods-10-00438-s001.zip › ╡lvarez et al. Figure 1.pptx]

## Slide 1
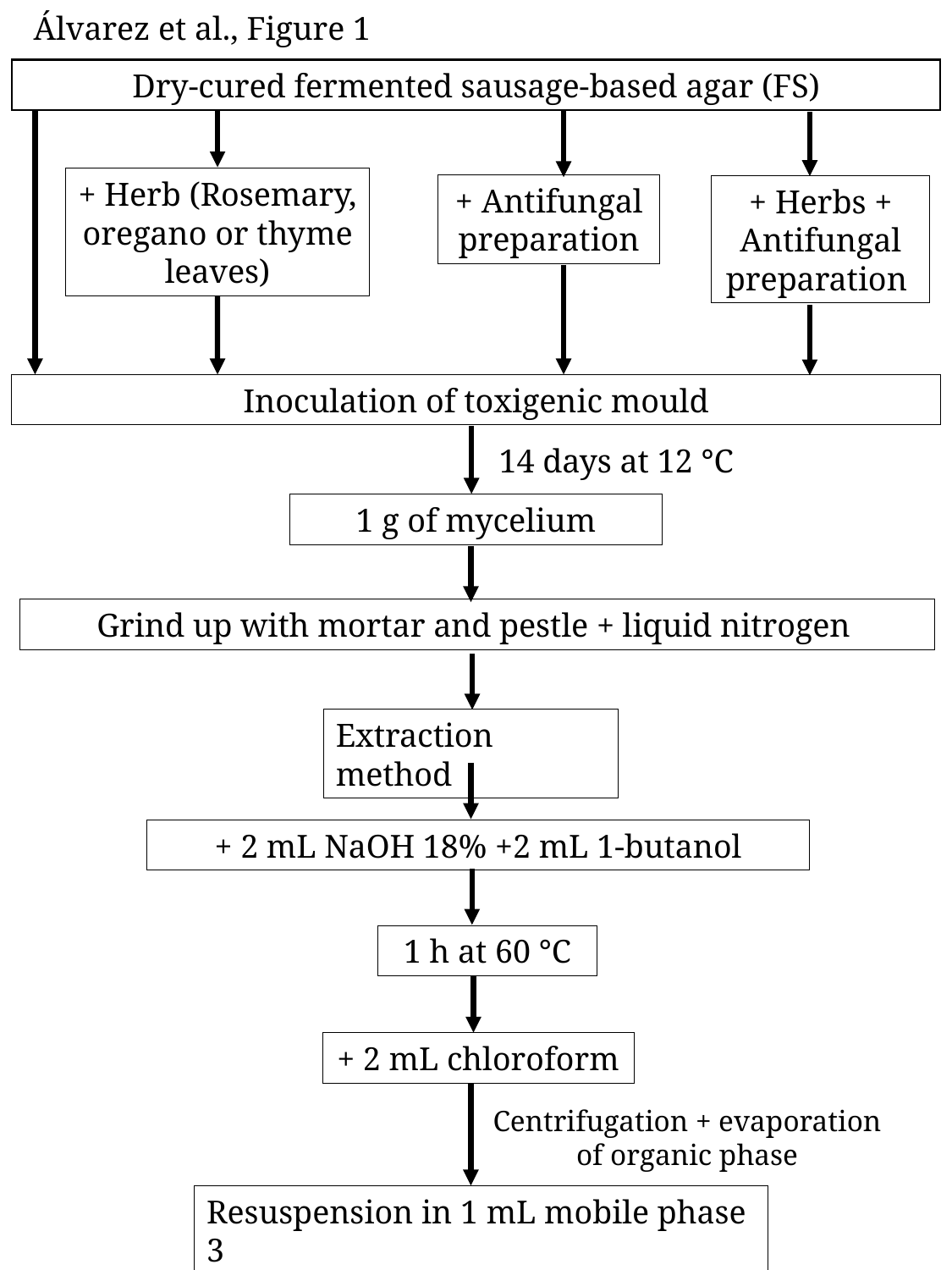

Álvarez et al., Figure 1
Dry-cured fermented sausage-based agar (FS)
+ Herb (Rosemary, oregano or thyme leaves)
+ Antifungal preparation
+ Herbs + Antifungal preparation
Inoculation of toxigenic mould
14 days at 12 °C
1 g of mycelium
Grind up with mortar and pestle + liquid nitrogen
Extraction method
+ 2 mL NaOH 18% +2 mL 1-butanol
1 h at 60 °C
+ 2 mL chloroform
Centrifugation + evaporation of organic phase
Resuspension in 1 mL mobile phase 3
