# Supplementary figures and images for: Development of a Methodology for Estimating the Ergosterol in Meat Product-Borne Toxigenic Moulds to Evaluate Antifungal Agents
Source: Foods. 2021 Feb 17;10(2):438. doi: 10.3390/foods10020438 (PMC7922909; doi:10.3390/foods10020438)

## Slide 1
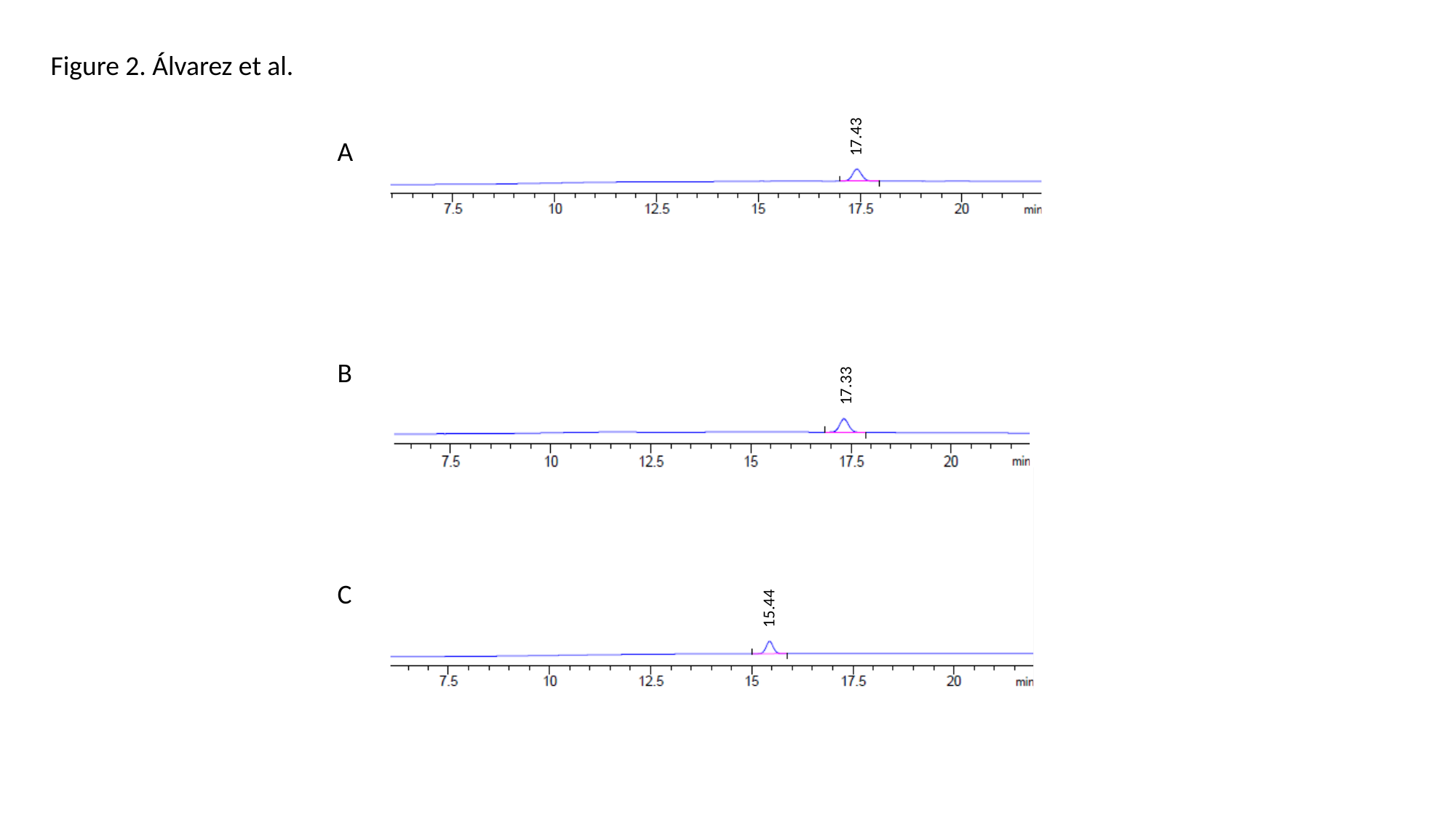

Figure 2. Álvarez et al.
17.43
A
B
17.33
C
15.44

Supplement: Supplementary file 1 [file foods-10-00438-s001.zip › ╡lvarez et al. Figure 2.pptx]

## Slide 1
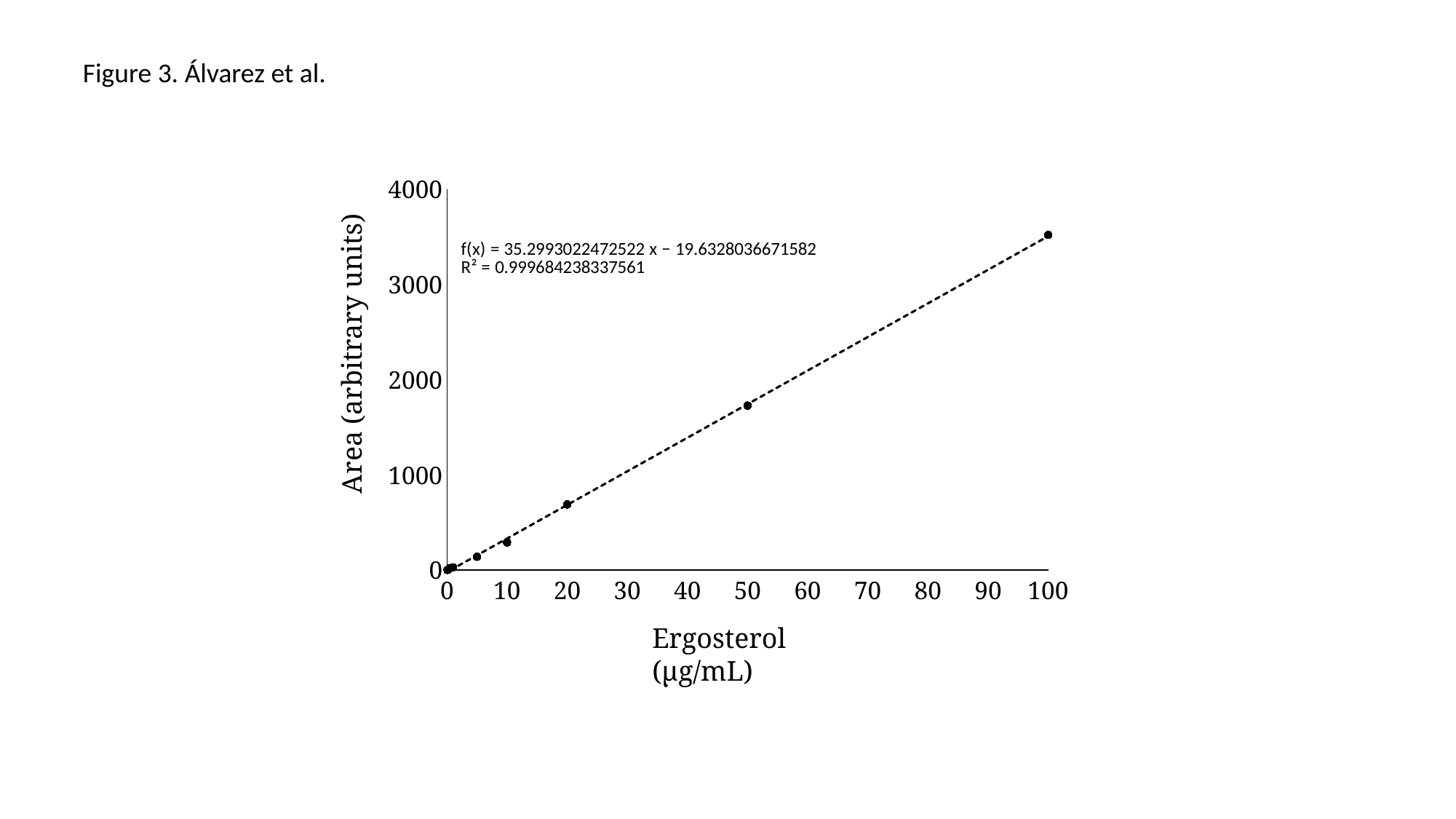

Figure 3. Álvarez et al.
### Chart
| Category | |
|---|---|Area (arbitrary units)
Ergosterol (µg/mL)

Supplement: Supplementary file 1 [file foods-10-00438-s001.zip › Alvarez et al. Figure 3.pptx]
